# The “pandemic” increase in lung ultrasound use in response to Covid-19: can we complement computed tomography findings? A narrative review

**DOI:** 10.1186/s13089-020-00185-4

**Published:** 2020-08-17

**Authors:** Luigi Vetrugno, Marco Baciarello, Elena Bignami, Andrea Bonetti, Francesco Saturno, Daniele Orso, Rossano Girometti, Lorenzo Cereser, Tiziana Bove

**Affiliations:** 1 Anesthesia and Intensive Care Clinic, Department of Medicine, University of Udine, University Hospital S. Maria della Misericordia, Udine, Italy; 2 Anesthesia, Critical Care and Pain Medicine Unit, Department of Medicine and Surgery, University of Parma, Viale Gramsci, 14, 431236 Parma, Italy; 3 Institute of Radiology, Department of Medicine, University of Udine, University Hospital S. Maria della Misericordia, Udine, Italy

**Keywords:** COVID-19, SARS-CoV-2, Ultrasonography, Lung, Pneumonia, Respiratory distress syndrome, adult, Severe acute respiratory syndrome, Multidetector computed tomography

## Abstract

Coronavirus disease of 2019 (COVID-19) is a highly infectious disease caused by severe acute respiratory syndrome coronavirus 2 (SARS-CoV-2), which has rapidly spread to a global pandemic in March 2020. This emergency condition has been putting a severe strain on healthcare systems worldwide, and a prompt, dynamic response is instrumental in its management. While a definite diagnosis is based on microbiological evidence, the relationship between lung ultrasound (LU) and high-resolution computed tomography (HRCT) in the diagnosis and management of COVID-19 is less clear. Lung ultrasound is a point-of-care imaging tool that proved to be useful in the identification and severity assessment of different pulmonary conditions, particularly in the setting of emergency and critical care patients in intensive care units; HRCT of the thorax is regarded as the mainstay of imaging evaluation of lung disorders, enabling characterization and quantification of pulmonary involvement. Aims of this review are to describe LU and chest HRCT main imaging features of COVID-19 pneumonia, and to provide state-of-the-art insights regarding the integrated role of these techniques in the clinical decision-making process of patients affected by this infectious disease.

## Introduction

In the ongoing battle against the disease caused by SARS-CoV-2 (COVID-19), which first broke out in the city of Wuhan, China, in December 2019, lung ultrasound (LU) [1] has quickly been recognized as a useful tool in the diagnosis and monitoring of the disease [2]. Ultrasound cannot replace lung computed tomography (CT) when examining the whole lung to choose a ventilator strategy [3], but point-of-care ultrasound (POCUS) may complement CT thanks to ease of access and general safety.

In this review, we summarize the main LU findings in COVID-19 pneumonia next to the equivalent CT signs [4], in the hopes that a correlation can be established to facilitate patient care. In clinical practice, the two imaging modalities can be integrated with great benefits for both patients and clinicians. Although limited in the extent of lung tissue it can explore, LU is particularly advantageous when confronting a COVID-19 outbreak: for one, ventilated patients need to be transferred to a CT suite, which can be outright dangerous in the face of severely impaired gas exchange commonly seen in COVID-19 intensive care unit (ICU) patients. We are not yet aware of LU signs specific for COVID-19, but signs of interstitial pneumonia are well recognized and have been found to be applicable in these cases, with some exhibiting high sensitivity [5, 6]. Additionally, it is now sufficiently clear that LU outperforms plain chest radiography in terms of sensitivity for acute respiratory symptoms in critical illness [7, 8].

A reasonable approach may be to perform CT scans on ICU admission to finalize diagnosis and to better inform the choice of ventilation (lung phenotyping) [3]; in order to reduce radiation and viral exposure, it could be repeated after a reasonable interval of time for prognostic information, whereas POCUS could be used to reduce ionizing radiation load and device contamination in many situations where it has been shown to be superior to chest x-ray and (in selected cases) as useful as CT [9]. Another setting in which LU is becoming essential is that of community outreach by specialized teams screening residents of nursing homes and long-term care facilities, which may have acted as a reservoir for SARS-CoV-2 [10]. Conversely, LU may not explore the entirety of the organ, and one should always consider CT when point-of-care monitoring fails to explain the current clinical respiratory status, especially in COVID-19, where thrombotic complications are increasingly found to be pivotal in the disease [11]. The aim of this review is thus to help physicians become familiar with LU and CT imaging in COVID-19.

### Lung ultrasound and COVID-19

**Fig. 1:**
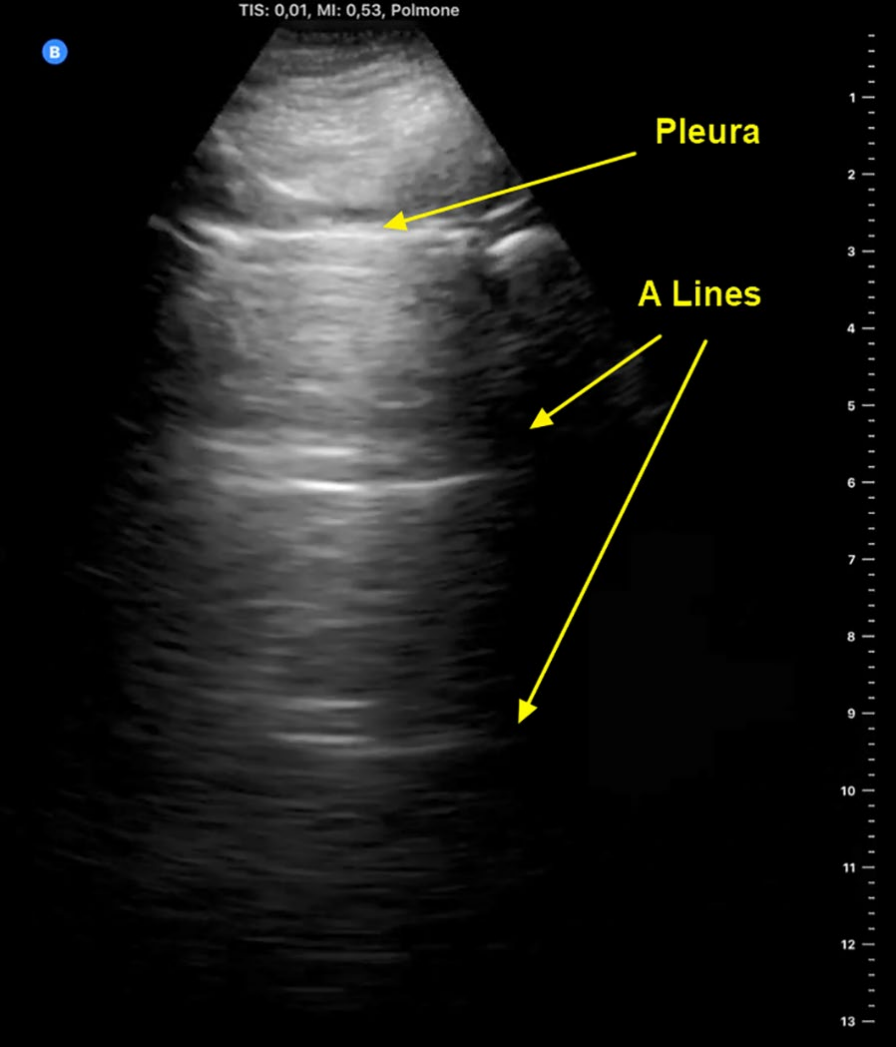
Anterior parasagittal lung ultrasound scan of the thorax. The parietal and visceral pleura appear as a hyperechoic line with sliding movements (also see Additional file 1: Clip S1). A lines are thought to be mirror artifacts from the near-total ultrasound reflection by the pleura-alveolar gas interface

Patients with interstitial syndromes usually display two types of artifacts. A-lines are the ‘default’ artifacts in healthy lung tissue, caused by soundwaves reverberating between pleura and the transducer, as the air-tissue interface reflects almost all the ultrasound packets. The sliding motion of the hyperechoic line in the near field depicts the movement of visceral over parietal pleural. The presence of A-lines and lung sliding signifies normal lung aeration (Fig. 1 and Additional file 1: Clip S1) [12].

**Fig. 2:**
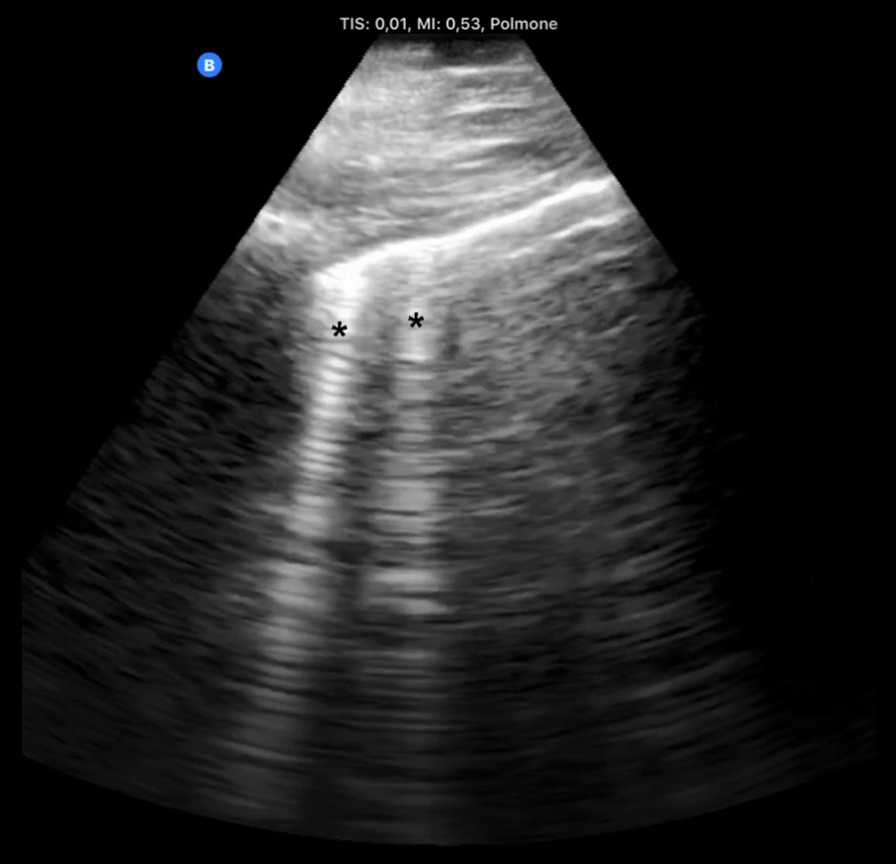
B-lines. Hyperechoic artifacts descending from the pleural line to the bottom of the screen are called “B lines” (black asterisks); by definition, a B line obscures any A line along its path. Two B lines per scan field are considered normal; ≥ 3 lines are associated with varying degrees of interstitial syndrome

B-lines are hyperechoic artifacts arising from single points along the pleural line and projecting to the bottom of the screen. They are thought to originate from locally increased tissue density which enhances reverberation from alveoli and distal airways [9] (Fig. 2 and Additional file 2: Clip S2).

The clinical significance of B-lines depends mostly on quality and quantity: **≤ **2 B-lines per intercostal space, interspersing A-line, and a sliding pleural line rule out pneumothorax and are considered normal variation. Three or more well-spaced B-lines (a “B-profile”) are associated with interstitial syndromes [12, 13].

**Fig. 3:**
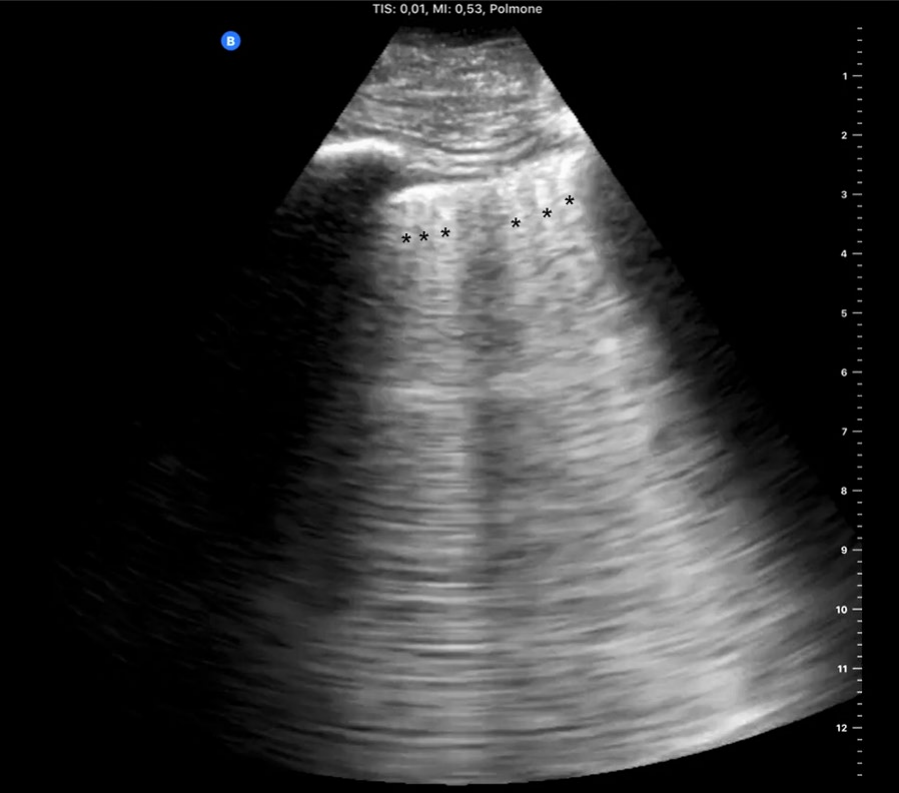
Confluent B-lines. Multiple confluent B-lines (asterisks) and occupying ≥ 50% of the field of view define the “rocket launch” sign or “glass rockets.” This is associated with more overt interstitial syndrome. Additional file 1: Clip S1: Anterior parasagittal scan of the thorax. The parietal and visceral pleura appear as a hyperechoic line. Pleural sheaths are seen sliding with respiration

**Fig. 4:**
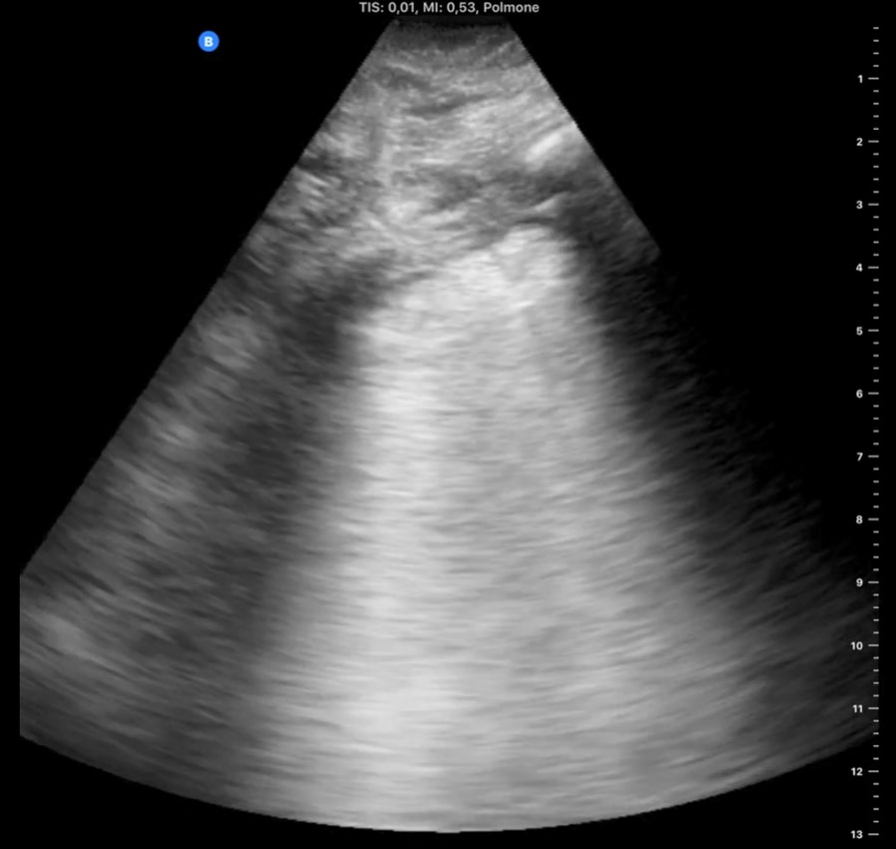
White lung. In this parasagittal scan, the whole intercostal space is occupied by continuous hyperechoic artifact commonly referred to as “white lung.” The pleural line also appears coarser and thicker than normal. This image is associated with advanced interstitial syndrome and may precede complete loss of aeration (consolidation)

The pleural line is normally thin and bright but, when inflamed, it can become thickened and less mobile, with B-lines arising from it and arcing slowly. The pleural line can also be interrupted by lung consolidation and loss of aeration (the “shred sign”) [14]. At the beginning of lung involvement in COVID-19, B-lines (with lung sliding) are distributed heterogeneously between spared regions of A-lines (Fig. 3). As the disease progresses, lung density increases, as does the amount of B-lines, leading to coalescence of the artifacts into “glass-rockets” and then “white lung” (Figs. 3 and 4, respectively; see also Additional file 3: Clip S3 and Additional file 4: Clip S4), equivalent ground-glass opacities (GGO) in CT scans [1]. Amid a pandemic with a very high prevalence of the disease, high density of B-lines with spared areas and lung sliding plus fever and cough is high suspicious for COVID-19 infection, and these patients should be managed accordingly [15, 16]. Glass rockets or white lung are typically associated with impaired oxygenation requiring supplemental oxygen.

**Fig. 5:**
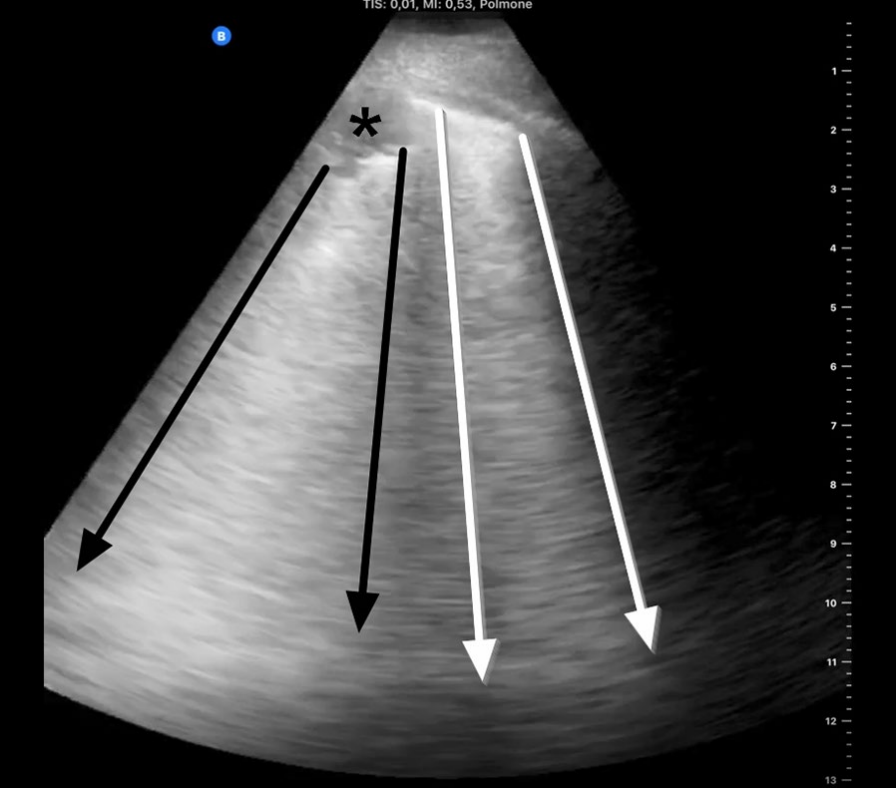
Subpleural consolidations and “light beam”. Two artifacts frequently seen in COVID-19 with significant respiratory symptoms: small subpleural consolidation (asterisk), with white lung-like artifact projecting from its interface with aerated lung (between black arrows); and a “light beam”, a hyperechoic artifact (between white arrows), also similar in quality to white lung, but arising from a discrete region of normal pleura. The light beam is so called also because it is “turned off and on” (i.e., disappears and reappears) repeatedly, even in a single respiratory cycle

Finally, in advanced disease, lung density further increases as alveoli fill up with inflammatory cells and Lung ultrasound findings in patients with COVID-19 pneumonia byproducts, with loss of aeration and a corresponding LU aspect called “tissue-like” (Fig. 5). This is equivalent to consolidation as seen in CT scans. The appearance of consolidations in LU is similar to that of liver tissue, with hyperechoic inclusions corresponding to distal bronchi which may or may not be filled with secretions [14].

### What is new in this context?

Recently, Volpicelli et al. have described some aspects of B-lines that seem to increase the diagnostic probability in patients suspected of COVID-19 pneumonia [5], in combination with clinical phenotype, blood tests and nasal-pharyngeal swab. They report on a sonographic sign named “light beam” and described as a shining vertical band artifact arising from a wide portion of a regular pleura, as opposed to the discrete points of origin of B-lines. The artifact is thought to be present in the earlier phase of viral spread in the peripheral lung and associated with the early GGO alterations visible at CT scan. This sign has also been described by Huang et al. in patients with COVID-19 infection as a “waterfall” sign [17]. In COVID-19, the light beam artifact is typically interspersed among areas of normal lung aeration or discrete B-lines, often alternating with areas of pleural line irregularity and peripheral consolidations. These signs identify affected regions, with patchy distribution in the two lungs [17–19] (Additional file 5: Clip S5).

In COVID-19, two pathological phenotypes have been described. One is the L-type, with normal or minimally reduced respiratory system compliance; these patients have been dubbed by some Authors as “happy hypoxic”, and a prospective multicenter study is currently investigating whether the light beam sign could be an early sign of disease in these cases. On CT, they show diffuse infiltrates but a low margin of recruitability (hence the name), defined as a relatively minor volume of de-aerated lung tissue [3]. The H-phenotype is characterized by low- to very low respiratory system compliance with high potential for recruitment with higher mean airway pressures; CT of these patients typically shows extensive consolidation with or without superimposed bacterial pneumonia. While L-type COVID-19 is intuitively characterized by the presence of LU signs such as those we described above (a multicenter study is ongoing to confirm this hypothesis), LU of H-type COVID-19 might have more in common with bacterial pneumonia and atelectasis. On CT, up to three different profiles can be described according to the distribution of consolidations [20].

From their characteristics, it has been proposed that different phenotypes require distinct respiratory management strategies, based on recruitment potential [3]. However, not all experts concur with this view, promoting instead the concept of a continuous disease which may worsen with mechanical ventilation itself and/or opportunistic infection [21].

### What is known about LU?

Certain characteristics of B-lines can help rule out COVID-19 infection, for example when B-lines are unilateral, which is more typical of initial pneumonia of likely bacterial origin, although underlying COVID-19 pneumonia cannot be ruled out [12]: theoretically, H-type COVID-19 pneumonia might share these features on LU. A unilateral consolidation within a large pleural effusion suggests bacterial pneumonia rather than COVID-19 pneumonia [22]. Pleural effusion is a well-known sign that appears as a hypo- or anechoic space in LU, distributed in gravity-dependent fashion [23, 24]. Pleural effusion is easy to see, and it may be possible to be estimate its volume with simple calculations (Balik formula) [22]. Sometimes septa can be seen floating in effusions, which is more typical of bacterial or fungal infection. However, in COVID-19, we have observed that large pleural effusions are rarely present and an alternative diagnosis, or superimposed infection, should be considered when confronted with such sign.

### Lung ultrasound score (LUS)

**Fig. 6:**
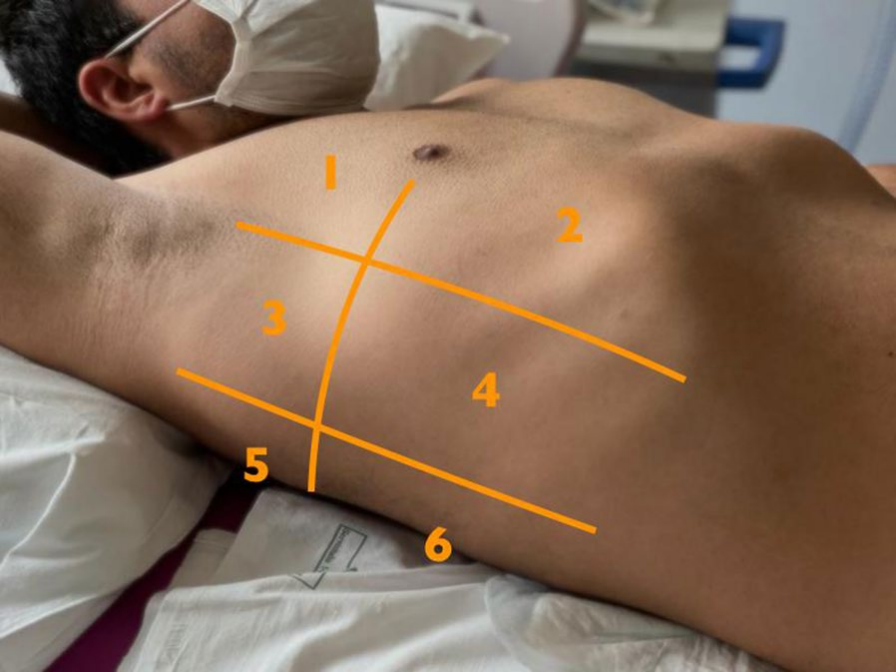
Representation of the 6-zone model for lung ultrasound score (see Ref. [21]). The parasagittal lines are the midclavicular, anterior and posterior axillary lines; the transverse line is at the 4th intercostal space, roughly equivalent to hilar lines

When performing LU, it is customary to divide the chest into 8 to 16 regions. We will describe an approach where the chest is divided into 12, defined by the intersections of the anterior and posterior axillary lines with a transverse plane passing through both nipples (Fig. 6). These regions can be scanned with different probes, depending on personal experience and preference, the most common being convex low-frequency and phased array (echocardiography) transducers [25].

Lung ultrasound score is a system in which each of 12 lung zones is assigned points, which are then added up [26]. One point is assigned to zones with A-lines and pleural sliding, or ≤ 2 B-lines; 1 point is for ≥ 3 well-spaced B-lines (interstitial syndrome); and 2 points are calculated for coalescent B-lines or “glass-rockets”—equivalent to GGO in CT; finally, consolidation with “tissue like-sign” has a score of 3. Therefore, the total score may range between 0 (normal lungs) and 36, in the worst-case scenario of completely de-aerated lungs [25]. We do not know how the “light beam” sign or small superficial lung consolidations may affect this score.

### Diagnostic performance of LU

Ultrasound distinction between acute cardiogenic pulmonary edema and acute respiratory distress syndrome (ARDS) in a critically ill patient can be difficult [27]. As is the case in every interstitial syndrome, non-infectious causes of pulmonary edema are always a differential diagnosis [28, 29].

The presence of bilateral B lines is associated with an interstitial syndrome, as previously reported [30], but it is poorly specific (while sensitivity is close to 100%). On the one hand, the presence of some ultrasound signs such as abnormalities of the pleural line, the reduction of pleural sliding, interspersed sparing areas, and the presence of consolidations can suggests ARDS over cardiogenic pulmonary edema (sensitivity 83–100% and specificity 45–100%) [29]. On the other hand, evidence on the diagnostic value of LU in ARDS is still very discordant. Diagnostic accuracy of LU for interstitial syndrome suffers considerably from variability between different research groups [area under the curve (AUC) of the receiver operating characteristic curve (ROC) from 85 to 95%] [31, 32]. The diagnosis of pneumonia by detection of lung consolidations (typical of bacterial pneumonia) was found to have sensitivity and specificity approaching those of CT (up to 95% and 91%, respectively). Ultimately, the diagnostic accuracy of LU seems to be greater than 90% [24, 25]. In viral pneumonia, LU has shown good sensitivity (94.1%) but inferior specificity (84.8%), with positive and negative predictive values of 86.5% and 93.3%, respectively [33]. Again, signs of interstitial syndromes can be present in a host of different conditions. [34], and at the time of this writing only anecdotal reports have been published for LU diagnosis of COVID-19.

## Chest CT and COVID-19

### Imaging features

The spectrum of chest CT findings encountered in viral pneumonias reflects variable combinations of a few histopathologic features (i.e., diffuse alveolar damage, intra-alveolar hemorrhage, and interstitial inflammatory cell infiltration), and include: (i) mosaic attenuation pattern (caused by hypoventilation of alveoli distal to bronchiolar obstruction and secondary vasoconstriction of the same areas); (ii) ground-glass opacity (GGO) and consolidation (related to the coexistence of airspaces partial filling and thickening of interstitial tissue); (iii) nodules, micronodules, and tree-in-bud opacities (indicative of centrilobular bronchioles that are dilated and impacted with mucus or fluid, along with peribronchiolar interstitial inflammation, infiltration, or fibrosis); (iv) interlobular septal thickening (due to the presence of interstitial fluid, cellular infiltration, or fibrosis); and (v) bronchial and/or bronchiolar wall thickening (due to inflammation or fibrosis) [35].

**Fig. 7:**
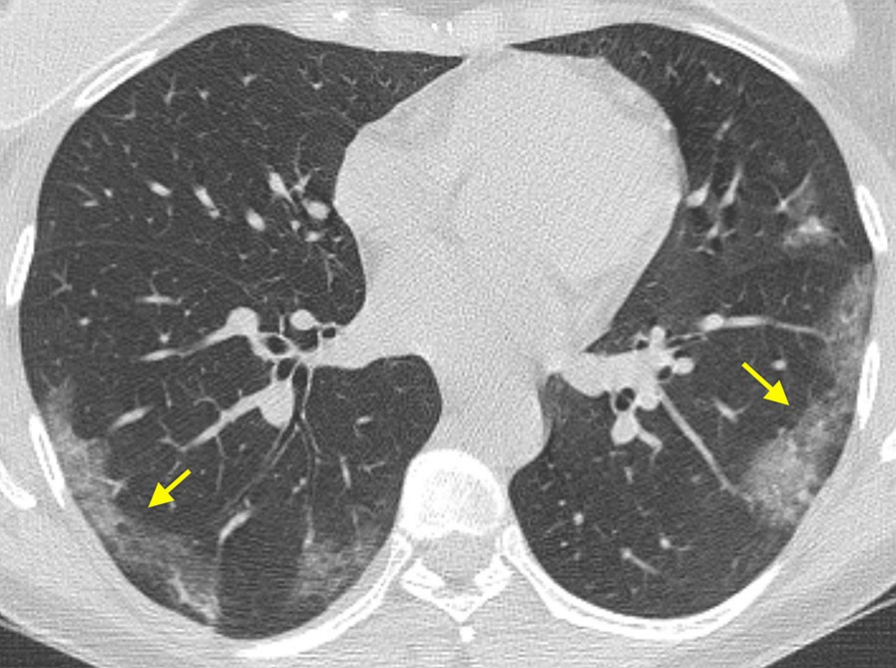
Typical chest CT imaging appearance of COVID-19 pneumonia. High-resolution CT image of a female COVID-19 patient presenting fever with cough for 5 days shows bilateral, multifocal, and peripheral areas of pure ground-glass opacity (GGO) (arrows)

**Fig. 8:**
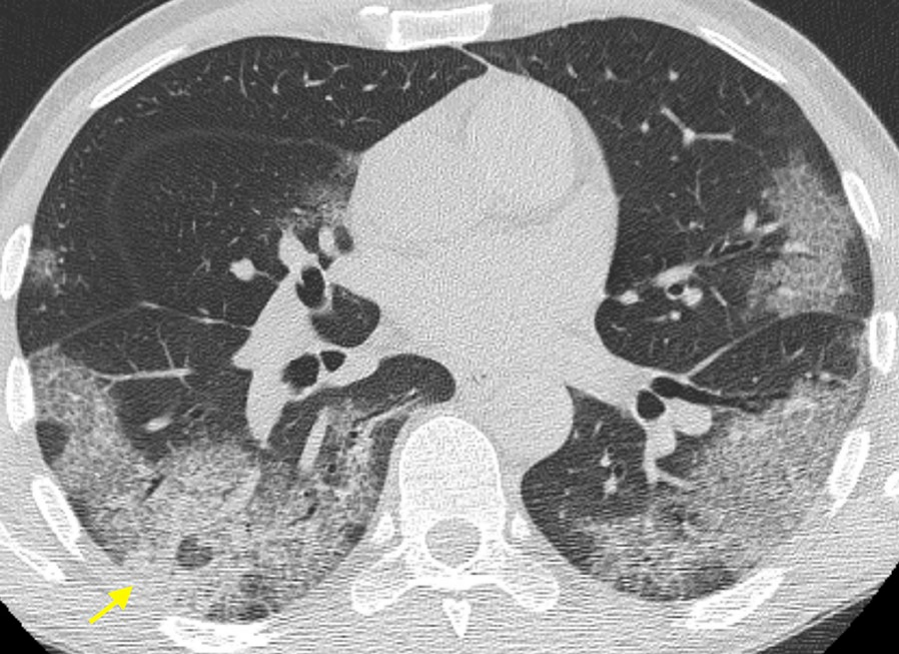
Typical chest CT imaging appearance of COVID-19 pneumonia. High-resolution CT image of a COVID-19 patient presenting fever with dry cough and myalgia for 10 days shows bilateral, multifocal, peripheral, and confluent areas of ground-glass opacity (GGO) with “crazy-paving” pattern, along with perilobular consolidation in the right lower lobe (arrow)

**Fig. 9:**
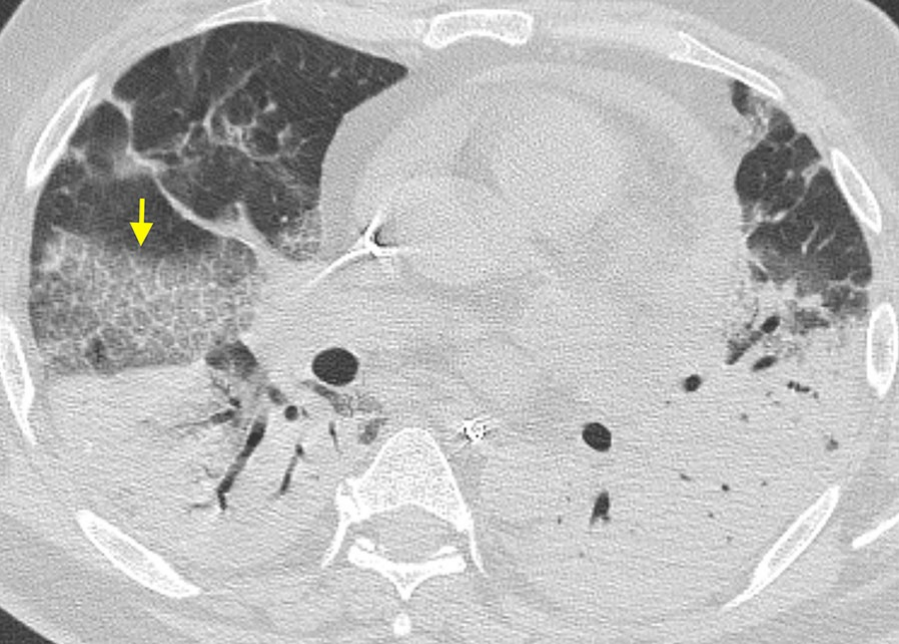
Chest CT imaging appearance of severe COVID-19 pneumonia and acute respiratory distress syndrome (ARDS). High-resolution CT image of a COVID-19 patient admitted to intensive care unit shows bilateral areas of consolidation predominantly affecting the dependent areas of both lungs, along with ground-glass opacity (GGO) with “crazy-paving” pattern in the right middle lobe (arrow)

As a general rule, all the viruses of the same family causing pneumonia share the main pathological changes and, consequently, show similar CT findings [36]. For this reason, pneumonias due to SARS-CoV-2 show HRCT features that resemble SARS-CoV and MERS-CoV pneumonias (all viruses belonging to Coronaviridae family), caused by injury to pulmonary epithelial cells with hyaline membrane formation in the alveoli, along with proliferative fibrous tissue blocking alveoli and small airways, and interstitial inflammatory infiltrates (mainly lymphocytes) [36, 37]. These pathologic alterations are supposed to be the causes of most typical CT findings of COVID-19 pneumonia, namely GGO and consolidation, with a prevalence range of 36–86% for GGO alone, and of 36–64% for the combination of GGO and consolidation. Their distribution is usually bilateral (87%), peripheral (in 56–97% of cases) and multifocal (76%), often with lower lung predominance (54%) [38–41] (Fig. 7). GGO may have round morphology and, in up to 5–36% of cases, configures a “crazy-paving” pattern (defined as thickened interlobular septa and intralobular lines superimposed on a background of GGO, resembling irregularly shaped paving stones; see Fig. 8) [42–44]. The prevalence of consolidation tends to increase later in the course of the disease, possibly reflecting an inflammatory reaction of the lung tissue (Fig. 9) [40, 43].

The presence of GGO and/or consolidation with a typical pattern of distribution in patients with high clinical likelihood of disease helps consolidating the suspicion of COVID-19 pneumonia in clinically borderline cases (e.g., when the results of RT-PCR are still unavailable on initially negative) [45].

**Fig. 10:**
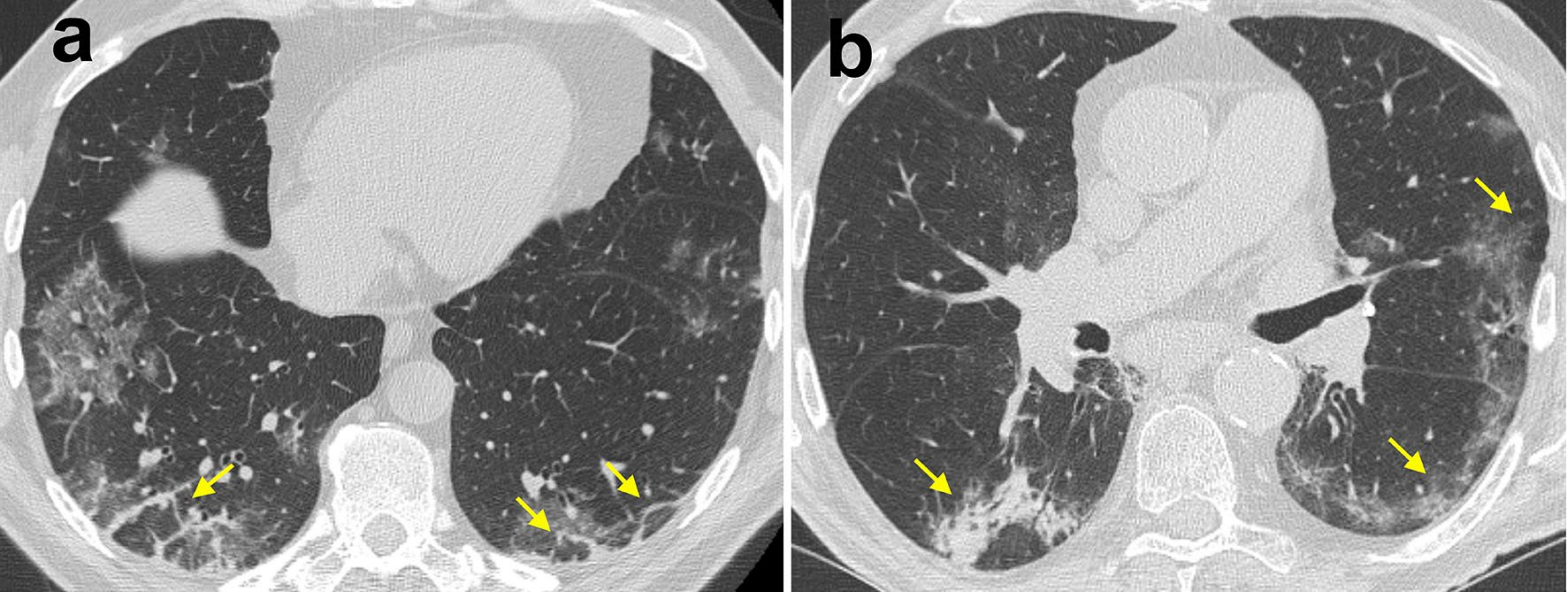
Organizing pneumonia pattern of lung injury in COVID-19 pneumonia. High-resolution CT images of a male COVID-19 patient presenting fever with cough and dyspnea for 15 days (**a**) and of a different male COVID-19 patient 14 days after the clinical onset (**b**) show perilobular thickening and parenchymal opacities (both ground-glass opacity and consolidation), along with superimposed signs of distortion, in the posterior subpleural regions of both lower lobes (arrows)

Other CT findings have been reported in COVID-19 pneumonia, including: air bronchogram (in up to 90%), bronchial wall thickening (in up to 30%), bronchus deformation (in up to 32%), pulmonary nodules (in up to 23%, mainly centrilobular, with or without tree-in-bud pattern), bubble-like air-containing spaces (in 80% of advanced-stage disease cases), and dilatation of pulmonary vessels around and/or within the lesions (in almost half cases) (Fig. 10) [38, 40, 46].

Those findings are of limited significance in patients with confirmed diagnosis and presence of GGO and consolidation. When presenting alone in less defined clinical situations (e.g., patients with negative RT-PCR results), they can act as confounders in imaging interpretation, or suggest alternate diagnosis.

**Fig. 11:**
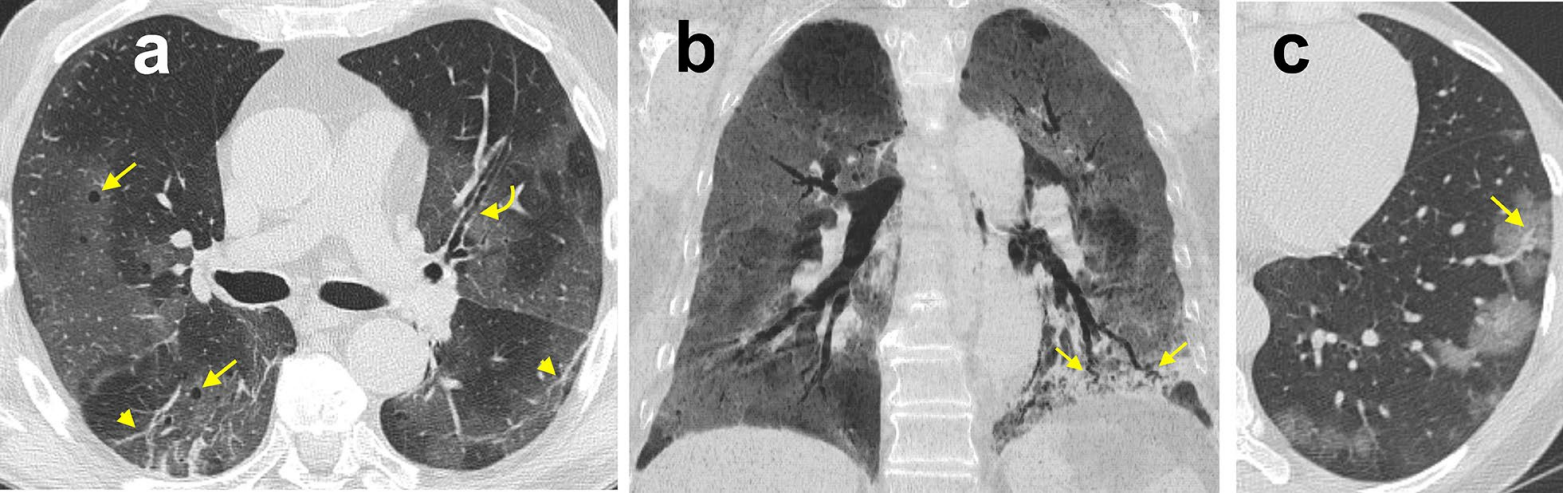
Ancillary chest CT imaging findings in COVID-19 pneumonia. **a** High-resolution CT image of a 73-year-old male COVID-19 patient presenting fever with dry cough for 5 days shows bilateral, multifocal, peripheral areas of ground-glass opacity (GGO) with mosaic appearance, along with superimposed fibrous stripes in the lower lobes (arrowheads), bronchial wall thickening (curved arrow) and bubble-like air-containing spaces (straight arrows). **b** 8-mm Min-IP coronal CT image of a 73-year-old female COVID-19 patient presenting fever for 5 days shows bilateral, multifocal, peripheral GGO, along with consolidation in the left lower lobe presenting with air bronchogram and bronchus deformation (arrows). **c** High-resolution CT image of the same patient as inset (**a**) shows dilatation of pulmonary vessels within one of the multiple GGO in the left lower lobe (arrow)

Of note, GGO and consolidation in COVID-19 pneumonia can reflect an organizing pneumonia (OP) pattern [47]; OP is usually part of the normal repair process of an acute or subacute lung injury [e.g., diffuse alveolar damage, (DAD)], mainly infectious in origin, displaying characteristic plugs of fibroblasts and myofibroblasts that organize both in airspaces and interstitial tissue [48]. In COVID-19 pneumonia chest CT may show certain features that, albeit nonspecific, may suggest coexistence of DAD in its fibrotic phase and OP pattern of lung injury (e.g., reverse halo sign, consolidation with perilobular pattern, peribronchial or peribronchiolar nodules) (Fig. 11). It has been supposed that the pathological pathway of COVID-19 pneumonia is sustained by two consecutive and overlapping stages, triggered by the virus itself in the earlier phase, and by the host response in the second phase [49]. Consequently, when chest CT is performed in patients with clinical signs of viral pneumonia, pulmonary findings presumably reflect a combination of concurrent viral and host inflammatory response. One may argue that, in case of predominant OP features, chest CT may hint at a self-reinforcing systemic inflammatory process, thus potentially suggesting the use of immunosuppressant drugs.

In order to aid radiologists in recognizing CT findings of COVID-19 pneumonia and improving their communication with referring clinicians, the Radiological Society of North America (RSNA) has grouped the above-described CT findings into four categories (typical, indeterminate, atypical or negative), according to their reported incidence in COVID-19 pneumonia [50]. However, radiologists should always keep in mind that patients who have underlying lung pathologies (e.g., extensive emphysema, lung fibrosis), or are immunocompromised might present with atypical chest CT pattern, thus reducing the diagnostic confidence [51, 52].

### Diagnostic performance and role in clinical practice

The role of imaging in the clinical decision-making of COVID-19 should be tailored depending on locally available resources, with the aim of assisting referring physicians in clinical decision-making, while guaranteeing the safety of healthcare professionals performing examinations, and minimizing the risk that themselves become vectors of infection [51, 53, 54].

A Multinational Consensus Statement from the Fleischner Society, involving a panel of experts in different fields (e.g., thoracic radiologists, pulmonologists, and anesthesiologists), stated that radiological imaging (plain x-ray or CT, depending on local resources and expertise): (i) has generally no role in patients with no symptoms or mild features of COVID-19; (ii) is advised when a patient present with moderate-to-severe features suggesting COVID-19 infection (regardless of COVID-19 testing results), since it can establish baseline pulmonary status, help in risk stratification for clinical worsening, assess complications (e.g., pulmonary embolism, superimposed bacterial pneumonia, or heart failure due to myocardial injury), or reveal alternative diagnoses explaining patient’s clinical features; (iii) can provide a presumptive diagnosis of COVID-19 for rapid triage and consequent decisions about clinical management and measures of epidemic control, when the pre-test probability of COVID-19 is high and the healthcare setting is resource-constrained (with potential limited availability of viral testing kits) [45].

**Fig. 12:**
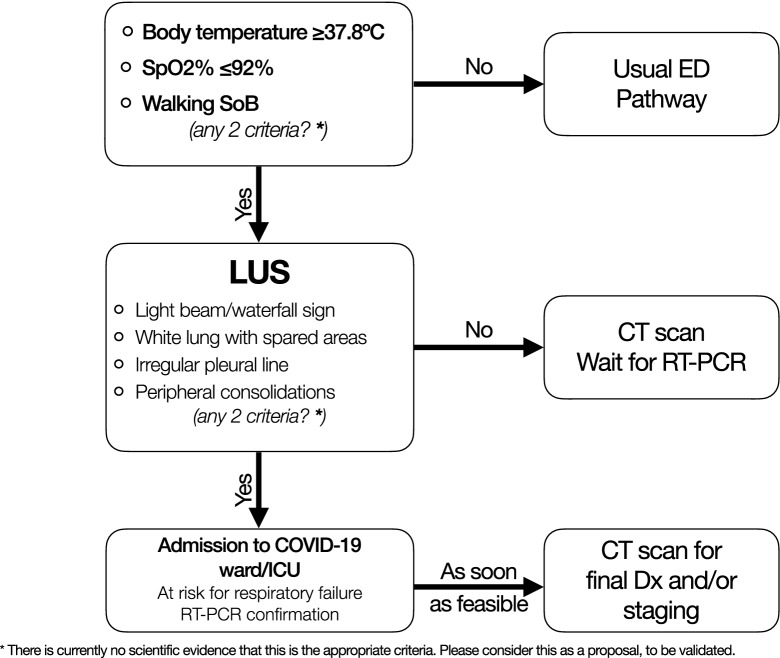
A proposal for a LU-based approach to triage and admission during outbreaks with high-volume influx of patients. There is not enough evidence, yet, to recommend specific LU criteria over others, but we hypothesize these may be the most useful ones. During resource constraint phases, feasibility and throughput volume can be maximized by the use of LU, followed by CT when appropriate. *LU* lung ultrasound, *CT* computed tomography, *ICU* intensive care unit, *RT-PCR* real-time polymerase chain reaction, *Dx* diagnosis

Concerning the latter scenario, a valuable complementary role for CT has been reported by Ai et al. [4] in a cohort of 1014 COVID-19 patients in Wuhan, Hubei, China. With RT-PCR as reference standard, sensitivity, specificity, positive predictive value (PPV), negative predictive value (NPV), and accuracy of chest CT in diagnosing COVID-19 were 97%, 25%, 65%, 83%, and 68%, respectively [4]. At the beginning of February 2020, the National Health Committee of China (NHC) established for Hubei Province the equivalence between COVID-19 RT-PCR-confirmed cases and clinically diagnosed cases, the latter identified only by means of clinical history, physical and laboratory findings, and “chest imaging revealing abnormalities”, preferably CT [55]. Nevertheless, such an approach may be less suitable in other scenarios, especially with lower prevalence of disease (i.e., lower pretest probability) [56]. According the Royal College of Radiologists “the CT appearances alone will not obviate the need for viral testing and should not be viewed as equivalent to or replacing this” [57]. Reconsidering the data expressed by Ai et al. [4], we may expect a PPV lower than 65%, implying that even more than 35% of patients with positive CT are actually *false* positive. Such an image-centric approach poses serious decision-making problems, since non-COVID patients hospitalized in a COVID-dedicated healthcare floor are at high risk to become infected soon, as highlighted in a communication by the Italian Society of Radiology to the members on March 26, 2020 (Sardanelli F, personal communication). In addition, chest CT can be negative for pneumonia in symptomatic patients with RT-PCR-confirmed COVID-19. As an example, in a cohort of cruise ship passengers and crewmembers, Inui et al. reported a 20% of symptomatic cases having no CT findings consistent with viral pneumonia [39]. Of note, according to the American College of Radiology (ACR) recommendations, “a normal CT should not dissuade a patient from being quarantined or provided other clinically indicated treatment when otherwise medically appropriate” [58]. Conversely, RT-PCR should be repeated whenever initially negative in the face of a chest CT suggestive of COVID-19 (Fig. 12) [59].

## Conclusion

In conclusion, chest CT in patients with COVID-19 has a high potential to give valuable help to clinicians, as long as it is used in accordance with existing guidelines and recommendations. Further studies, particularly on a radio-pathological correlation basis, are warranted in order to tailor the CT to different clinical scenarios.

In a recent interview, Dr. Roberto Cosentini, the head of the Emergency Department at the main hospital the Bergamo province of Italy (one of the most stricken by the epidemic), mentioned up to 60–80 COVID-19 admissions, mostly concentrated in afternoon hours. Such circumstances may force even major hospitals into resource constraints,^1^ and when that happens, it may be conceivable to sacrifice diagnostic specificity in exchange for increased sensitivity and patient throughput through triage units; experience at one of our institutions led to the proposal of a LU-based algorithm for faster admission of patients with pneumonia during a possible future COVID-19 outbreak (Fig. 12). In such emergency contexts, LU may temporarily be the most feasible imaging modality, by allowing high-throughput patient assessment without transportation throughout the hospital; its high sensitivity when screening patients in the admission area may avert the risk of inappropriate discharges (i.e., compensating but sick patients). In the ICU, daily monitoring including LU allows for early detection of deterioration and/or improvement, which may influence the decision to transfer patients to lower-intensity units, although a correlation between LU findings and disease evolution over time has not been established [60]. LU is an interdisciplinary tool that can be used throughout the acute care hospital, with a common language when treating (COVID-19) lung pathology. However, for this to happen, definitions and skills need to be shared, diffused and consolidated in prospective evidence.

## Supplementary information

**Additional file 1: Clip S1.** Anterior parasagittal scan of the thorax. From top to bottom, the first (most superficial) hyperechoic line is the pleural line, characterized by its sliding layers. Further down, multiple A lines “mirror” the pleural shape and move together with it as the transducer is slightly tilted and rocked.

**Additional file 2: Clip S2.** Anterior parasagittal scan of the thorax. Up to 3 B lines appear on screen throughout respiratory cycles (white asterisks). A lines are erased where B lines appear.

**Additional file 3: Clip S3.** Coalescing B-lines with distinct points of origin in the pleural line and moving with it, while filling ≥ 50% of the field of the view. This artifact can be described as a “rocket launch” or “glass rocket”. Note how A lines become visible again as B lines slide out of view with respiratory cycles (spared areas).

**Additional file 4: Clip S4.** White lung artifact arising from an irregular (“shredded”) pleural line, just next to a spared area of the lung. No single B line is detectable. Also note how the visceral pleura appears detached from the chest wall: a millimetric effusion (shown as a thin hypoechoic line just above the visceral pleura) is quite common in severe interstitial syndrome.

**Additional file 5: Clip S5.** Two artifacts frequently seen in COVID-19 with significant respiratory symptoms. A small subpleural consolidation, indicated by the white caret (’^’) projects a white-lung artifact from its interface with deeper aerated tissue. To the right, a similar artifact arising instead from normal pleura disappears an reappears multiple times: a “light beam”.

## Data Availability

Not applicable.

## Abbreviations

ARDS: Acute (or adult) respiratory distress syndrome
AUC: Area under the curve
CT: Computed tomography
DAD: Diffuse alveolar damage
GGO: Ground-glass opacity
HRCT: High-resolution computed tomography
ICU: Intensive care unit
LU: Lung ultrasound
NPV: Negative predictive value
OP: Organizing pneumonia
PPV: Positive predictive value
RT-PCR: Reverse-transcription polymerase chain reaction

## Footnotes

1 “Like an earthquake.” Interview with Dr. Roberto Cosentini. The Italian Society of Emergency Medicine Official Website. https://www.simeu.it/w/articoli/leggiArticolo/3977/leggi [in Italian]. Last accessed June 16, 2020.
